# Women’s injection drug practices in their own words: a qualitative study

**DOI:** 10.1186/s12954-015-0041-6

**Published:** 2015-03-07

**Authors:** Ellen Tuchman

**Affiliations:** Silver School of Social Work, New York University, 1 Washington Square North, Room 402, New York, NY 1003 USA

**Keywords:** Injection drug practices, Initiation, Self-injection, Women, Harm reduction, Syringe exchange, Gender differences, Qualitative

## Abstract

**Background:**

There are significant gender differences in injection drug practices and relative risks involved for women who inject drug compared with men. This qualitative study aims to explore the social, contextual, and behavioral dimensions of injecting practices among women who inject drugs.

**Methods:**

Participants were selected by purposive venue-based sampling from a syringe exchange program in 2012–2013. In-depth interviews were conducted with 26 women to elicit detailed perspectives regarding injection drug use practices and women-focused decision-making. All interviews were transcribed verbatim and analyzed with Atlas.ti.

**Results:**

Participant’s mean age was 43.2 years, 48% Caucasian, 36% African American, and 16% Latina, poorly educated, mostly single, and heroin self-injectors. Three themes emerged; a) transitioning from non-injection to injection drug use; b) patterns and variations of initiation to injecting; and c) shifting toward autonomy or reliance on others. Women were predominantly influenced to transition to injection drug use by other women with their claims that injecting was a way to curtail their daily drug expenditure. More than half the women received their first injection from another woman in their social network rather than a male sexual partner. Self-injecting women exhibited agency around the circumstances of injection safety and potential risks. Other women revealed that their inability to inject themselves could and did make them dependent on others for unsafe injection practices.

**Conclusions:**

The finding that many women were influenced to transition to injection drug use and receive the first injection from a woman is contrary to literature claims that male sexual partners introduce and initiate women to injection drug use. Self-injecting women possessed capacity to act in a way that produced the results they wanted, not sharing prepared drugs or injecting equipment. In stark contrast, women assisted with injections could and did make them vulnerable to unsafe injecting. Findings support early prevention strategies that discourage women’s transition from non-injection to injection and development of female peer-driven experiential interventions to dispel myths for non-injection women and to increase personal capability to self-inject for women who require assistance with injecting, to reduce injection-related harm.

## Introduction

Unsafe injecting drug use (IDU) is a significant factor in the HIV transmission dynamics of women who inject drugs (WIDUs). Different understandings of risk behavior or harm exist among them, and such differences are likely to depend on the social, contextual, and behavioral domains in which IDU occurs. A recent review on IDU and HIV found that, compared to men, WIDUs experience faster progression from first drug use to dependence, increased likelihood of injection-related problems, and higher levels of risky injection, sexual risk behaviors, rates of HIV, and significantly higher mortality rates [1-4].

IDU when compared with non-injection drug use comes with a myriad of additional risk factors for women, including risks associated with injecting partners and the use of unsterile syringes [5-9]. Other factors associated with HIV risk among WIDUs include: injecting cocaine more than once per day, engaging in unsafe sex with an injection drug using partner, and transactional sex work [4].

Research has identified several personal and environmental characteristics which act as predictors for initiation to IDU for women. Factors include engagement in sex work, non-injection drug use history, lifetime history of sexual abuse (particularly at a younger age), exposure to trauma and violence, social networks that include IDUs, family history of drug use, social disadvantage (homelessness, low socio-economic status), lifetime history of incarceration, delinquent behavior (truancy or running away), young age of first illicit drug use, early sexual experiences, and mental illness or suicide attempts [1].

The context of the first injection also affects the potential risks faced by WIDUs for the duration of their injection career. Variables such as where the injection takes place, who is present, where the drugs and equipment come from, and types of drugs used are all important risk factors. Women are more likely to inject in a social setting, less likely to inject alone, more likely to have their spouse or partner present, and more likely to have been in a group of mostly WIDUs [10-14].

Scarce research illustrates that women with injection partners differ from women who self-inject. Self-injecting women relish the independence of being able to choose when and where to have their drugs, which vein to inject into, and freedom from reliance on an injecting partner. Women injected by others experience lack of agency, fearful of causing harm to themselves, and injected in areas of their body they would not ordinarily choose out of an urgent need to alleviate withdrawal. As such, partners sometimes had a pervasive influence on women’s drug use and injection practices [15]. The practice of being injected by others is a social vector promoting a higher vulnerability to HIV among WIDUs [16]. Barriers for women to self-inject are also lack of knowledge of proper injection technique, loss of accessible veins, drug withdrawal and increased vulnerability to HIV/HCV infection within the context of intimate relationships [17].

Data consistently show that IDU is a highly gendered activity. WIDUs have substantially different needs and face higher risks of disease than do men who inject drugs. Given this difference, it is surprising that much of the literature on IDUs does not distinguish between men and women when discussing prevalence, needs, risks, and outcomes of IDU. This has led to a possible underrepresentation of the WIDUs perceptions of their injection practices and significant factors in their HIV transmission dynamics. There is a clear need for more systematic collection of data specifically with WIDUS.

Qualitative research of WIDUs is scant in the literature and was chosen for this study because it allowed in-depth exploration and elucidation of the unique contextual factors of WIDUs, specifically around the shift from non-injection to IDU and the factors coupled with “self” versus “assisted” IDU implications for HIV transmission. This study addresses this gap by exploring the social, contextual, and behavioral dimensions of injecting practices of WIDUs in a syringe exchange program (SEP).

## Methods

### Sampling and recruitment

Purposive venue-based sampling was used to select WIDUs from the Lower Eastside Harm Reduction Center’s SEP in New York City, between October 2012 and August 2013. The HRC mission is to reduce the spread of HIV/AIDS, HCV, and other drug-related harm among IDUs and the community by incorporating a spectrum of strategies such as syringe exchange. The SEP provide free sterile syringes and collect used syringes to reduce transmission of blood-borne pathogens.

A screening form was administered at the time of initial contact to determine study eligibility. Study inclusion included women, >18 years, IDU in the past month, and the ability to speak English. Interviewers observed visible injecting marks confirming injector status.

### Procedure

Twenty-six WIDUs were interviewed for the study. Participants were interviewed in-depth by two trained interviewers using a topic guide encouraging the participants’ discussion of injecting drug practices and decision-making. The interview guide emphasized the following topics: substance use, IDU practices, and injecting decision-making. The one-time, in-depth interviews lasted between 60 and 90 min, digitally recorded, transcribed verbatim, and entered into Atlas.ti for data analysis.

The study received ethical approval from the New York University Human Subjects Committee. All participants were consented, provided with a project summary sheet, and received $15 for their participation.

### Coding and grounded theory analyses

The research used a grounded theory approach to understand the concerns, actions, and behaviors of participants and to explain those patterns of behavior [18]. Procedures for coding and interpreting the transcripts were consistent with those of grounded theory [18,19]. Members of the study team independently coded four transcripts and met to develop an initial list of open codes. The author independently coded four additional transcripts, adding codes and refining the list. The codebook consisted of codes, code definition, exclusions, and a quotation as a code example. A final set of codes was complete by the ninth co-coded transcript. Using this code list, all transcripts were co-coded separately by the team members—all discrepancies were discussed until consensus was reached.

Selective coding and constant comparative analyses were conducted to yield core categories or themes [20]. Themes lacking sufficient grounding in the data and/or linkages to other themes were not included in the model. When the selective codes and core themes reached saturation, the final grounded theory model was developed. In keeping with grounded theory procedures, an audit trail was used to document analytic decisions [21].

## Results

### Participant characteristics

The sample consisted of 26 women, ranging from 22 to 63 years, with an average age of 43.2 years. The racial-ethnic composition was 48% Caucasian, 36% African American, and 16% Latina. The sample was poorly educated, less than half graduated high school, and the entire sample were unemployed. Sixty-four percent were single/never married. Six women reported childhood sexual or physical abuse by male family members.

Four WIDUs were not participants in the SEP but receiving services in the HRC. All participants reported a period of illicit non-injecting drug use prior to injecting. The mean age of initial drug use was 16.2 years (*r* = 9–29). First injection mean age was 23.8 years (*r* = 12–58). Most injected heroin, with a few cocaine and speedball injectors. Average duration of injecting drug use was 18.2 years. Sixty percent were identified as self-injectors, 20% as assisted injectors, and remaining women were reported self and assisted injections.

### Themes from the cross-case analyses

Three main themes emerged in the interviews with respect to the social, behavioral, and contextual domains of WIDU practices and risks: (a) transitioning from non-injection to IDU, (b) patterns and variations of initiation to injecting, (c) shifting toward autonomy or reliance on others. These themes, along with illustrative quotes from participants, will be discussed below.

### Theme 1: transitioning from non-injection to IDU

Reasons why women transitioned from non-injection to IDU at any given point in time were complex. The expense of sniffing or smoking drugs was the primary reason many decided to begin injecting. For many women, drug use was often a social network experience shared with friends, partners, and spouses. Most women expressed the importance and safety of using drugs around people they knew and trusted. In many cases, the decision to transition to IDU was influenced by a combination of factors.

Women non-injectors were advised by their peers about the benefits of transitioning from non-injection to IDU. Examples included the following: they would use less drugs, spend less money, and get a better, quicker high compared to sniffing. Prior to their first injection, it was a common belief among participants that injecting would be a way to get their expensive habit under control.

*It was gettin’ expensive by sniffin’ and so my friend said it holds you longer and you don’t have to be doin’ as much bags … by sniffin as injecting like they say, which is what’s true* (23).

For some women, the shift from intranasal to IDU to their first injection was based on her need to increase the high and desire to go along with others or fit in with IDU social network.

*I sniffed heroin at 21 years old. I was 23 and basically wasn’t feeling it at all anymore, and I was now with a crowd of shooters that looked at me like I had four eyes because I sniffed* (21).

It was common for experienced WIDUs to encourage their non-injecting friend to change the route of their administration to injection by explaining the benefits of IDU such as the better quicker high than the intranasal route of administration:

*Me and her was together one day and she was like “Why don’t you shoot it? It’ll hit you better and you’ll feel it much better”, and I was real scared and she was like “I’ll hit you, I’ll hit you, I’ll hit you.” That was the first time, she hit me, and I started shooting drugs* (1).

The transition to IDU was often motivated by curiosity and self-gratification. Women were not always passive in the transition process. Often, they had an active role in their shift to injecting. Some women stated that injecting for the first time was their own idea, curious about injection but unable to inject themselves. This woman wanted to inject heroin so badly that she “blackmailed” someone to inject her.

*I got curious…one night I was like I want to try it, and he’s like, absolutely not happening, and I’m like, listen let me tell you something. I’m the one that’s bringing it [heroin] back up here and you ain’t going to find anybody else who’s gonna do it who’s as young as I am, so either you turn me onto it or I go…He actually hit me before he hit himself the first time* (18).

### Theme 2: patterns and variations of initiation into drug injecting

Most often, the first injection was prepared and administered by someone they knew, usually through a well-established social network of IDUs. An important finding was that again, a WIDU who was a friend or relative injected more than half the women their first time. Most of the initiates had only one other person present at initiation and in a private indoor venue; home, apartment, or bathroom. None of the women were alone the first time they injected.

Experienced WIDUS very often served as instructors by explaining the injection process and in almost most cases, the injector prepared the drugs and injected themselves before injecting the other women. The skill of the injector was important to women receiving their first injection. A skillful injector was thought to be anyone that could inject them without any complication or marking. Some initiators would take their time and be gentle, careful and patient and others already high may miss or go through the vein.

This woman was already in withdrawal, experiencing symptoms and not fearful of being injected the first time. She expressed how good she felt after the injection and no longer in withdrawal and continued IDU following their first injection.

*So she explained to me how it works… she told me not to be scared. I wasn’t. I really was feeling sick. I needed to get straight. I saw how she did it, she tied me up with her belt and she told me to relax and then she put the needle and got the vein real fast and she told me that as soon as you…pushed the syringe back and you see the blood floating, that’s how you get the vein. That’s all it took, just one time. The experience was amazing. I instantly felt ok* (10).

Only two women in the study self injected their first time but after they observed another injector who served as their instructor, usually a female friend, acquaintance, or relative. They both thought they had good veins.

*Well, I injected myself. I watched somebody inject themself and since I had good veins, at the time, I had no problem injecting myself…they showed me how to do it and that was it* (24).

Conversely, a quarter of the women reported a male sexual partner, spouse, or friend initiated their transition to their first injection.

*At first I snorted for a couple of years. Then after that I was seeing how…I’m snorting five bags to their equal to two and they’re like stoned…I said to myself, you know what? I never stuck a needle in my arm.’ I didn’t like needles. My sexual partner did everything for me…injected me. The first time it was, ‘I’m home! This is it!’ And I never stopped from then* (26).

In most urban areas where rates of IDU are high (NYC), there are common neighborhood locales for purchasing and using drugs. Among the highest risk locales are “shooting galleries” or “safe houses” places where people go and inject and usually pay in order to be able to inject. Hit doctors are frequently male and known within the IDU community for their skill as injectors. Some women who required assistance with their first injection went to safe houses belonging to IDU friends who lived alone and would serve as a place for a group of close friends to inject together.

For this woman, being reassured by an experienced, knowledgeable hit doctor who blew on her skin as the needle was pushed in to reduce stinging when being injected. Often, these decent experiences of assisted injecting ensured dependency on the hitter and injecting at the same time.

*I was young, 13 and down the block was what you’d consider a crack house and it was really dirty in there and they had 10 cats. I sat on the couch…he had a ponytail and he was really cool, he kissed my arm, before he did it and, I looked away and he did it for me and it was quick* (15).

### Theme 3: shifting towards autonomy or reliance on others

Most women in this study identified as self-injectors. For these women, the shift to becoming a self-injector is related to many protective strategies; independence from other injectors, self-sufficiency, choice of body injection site, and control over the time, place, and relative safety of injecting practices.

After learning how to self-inject, this woman expressed a strong sense of independence and autonomy of her ability to learn how to self-inject.

*I learned how to hit myself. A lot of people say, ‘oh well I need somebody to hit me,’ not me. I close, I drew it, and I put it back in. I didn’t need nobody to help me draw up. I didn’t need nobody to help me do that no more because I do everything myself. Everything* (11).

This woman described the process of teaching herself to self-inject. Her ability to self-inject also instilled feelings of competence, independence, control of oneself, and prevention of harmful, noticeable scars on her body.

*First time I self-injected was in my hand, and I fucked it up, like twice. But you know, then I got the hang of it. I didn’t push it in. I knew it wasn’t in because the blood wasn’t registering…I have no track marks. I felt a hell of lot more independent and more in control. …. Now I can do it everyday* (18).

Another woman strongly described her choice to self-inject as a competent and safer practice.

*I watched somebody inject herself. I had good veins, at the time. I had no problem injecting myself. They showed me how to do it and that was it. I think it’s safer. I don’t want someone else injecting me. I can tell when I register. I shoot up in my own way. That’s when I made the choice. I chose to be a self-injector because I do it my way* (24).

These experiences of self-injectors described above sometimes triggered women to contemplate the intrinsic worth of self-injecting. Many of the women who were self-injectors expressed a sense of autonomy and control in utilizing the SEP as a protective and safer measure.

*I always use clean syringes from here, I shared one time in my life, and I was very nervous, I got an HIV test after that. I was very scared to get that test, cause, this person was a junkie, and dirty. I always carry cleans. If my friends didn’t have cleans, I gave them cleans. If I use the same one more than once, I made sure I knew it was mine, I had a little makeup case that I carried all my needles* (26).

However, several women did describe requiring assistance with injecting because they lacked knowledge, uncomfortable self-injecting, or small veins making self-injection complex. Women who were injected by others experienced a lack of autonomy, reliance on others, and fear of causing harm to themselves. People sometimes injected women in areas of their body, or, they were people they would not ordinarily choose out of an urgent need to alleviate withdrawal. These risks of being injected by others; transmission of blood-borne viruses, bacterial infections, damage to the circulatory system, and overdose were rampant among this group.

This woman appeared to be unaware regarding the potential risks associated with being injected by another, until after she realized she was infected with hepatitis C.

*I didn’t know how to hit myself. Then after a while she would do it for me, I would have to call her. I started having to go to the shooting gallery…I learned when they would hit you…That’s how I think that I contacted the hep C, you only get it blood to blood. Then, I really did wanted to learn how to hit myself, but I never did learn how to do that* (1).

An implicit understanding existed for some women that they should not question the injector’s skill or knowledge when being injected, for example, about which vein to inject into. Women injected by others were clearly much less in control of their injecting situation. Skillful and experienced injectors however did not always successfully inject others. Women had experienced harm from injectors and often compared the abilities of different injectors.

*He was injecting me. At first, because very, very hard for me to hit myself. I had these little girly veins. They roll. It’s really hard, so I used to let him hit me in my neck because I had bad veins. It’s really hard* (21).

Women requiring injecting assistance often spoke about being second on the needle. It was common for the women to receive their injection after the injector had self injected. The injector’s condition often affected the injection technique particularly if they had already self injected. Injectors being heavily intoxicated when injecting women placed them at an increased risk of physical harm.

Some women thought people who self injected before injecting them were selfish and impatient for drugs.

*I was asking do I need my own set? She would say no. She always went first and she was mainlining and did me second and it was actually hell because I always had to wait for her and then sometimes she didn’t clean the needle. [She] done die of AIDS* (8).

For others, experiencing withdrawal affected their ability to assess risks or take precautions. This woman describes how she just wanted to get high.

*I didn’t think about safety…I didn’t think about nothin’. All I wanted was to feel the heroin run through my veins. There was a needle that was shared and at the moment I don’t even think about it. I didn’t think about this person, how well do you know him or did somebody else use it and if he just cleaned it* (7).

Women’s experience of physical harm and damage to their veins as a result of being injected by others was common. Women spoke about the injector “missing” as a result of the needle not going into the vein or being pushed through the vein were frequent which often resulted from the injector rushing when injecting them due to their own withdrawal. The injector’s condition often affected the injection as they took less time and care when injecting the women if they had already self injected. Several participants described harmful and risky syringe-sharing behaviors with IDU partners who provided injection assistance:

*He would have to hit me cause I couldn’t hit myself. Sometimes we would get I into arguments over who would go first. We had this thing between us that we used to call it I say I’m first on the case. But if I call I’m first on the case first, he would get angry cause he had to hit me first. He would do it in a rough way* (13).

## Discussion

This qualitative study contributes to the knowledge concerning social, contextual, and behavioral domains affecting WIDUs injecting practices through three key themes; transitioning from non-injection drug use to injection, initiation to IDU, and shifting towards autonomy or reliance on others.

First, the transition process, from non-injection drug use to IDU, was greatly influenced by women’s social network. Myths and half-truths about IDU was spurred most often by WIDUs beliefs; IDU is a more efficient route of drug administration, a way to curtail daily drug expenditures, and a promise made more alluring by witnessing the amplified high female friends experienced after IDU. This finding is often ignored in both the prevention and treatment literature. But substance abuse treatment is a prospect for early intervention for women who do not inject drugs. An example may be a women’s group aimed to hinder transition to injection through heightening their awareness of the risks, outlining ways that women make the transition into injecting, and by encouraging the development of anti-injecting rationales and resistance skills.

Other women expressed the pressure of their network, which created an aspiration to be similar and fit in with their network, a network comprised mostly of WIDUs. This finding supports existing literature on the influence of peer pressure and the supposition that injectors are related to, not isolated from, the larger social group of injectors [14]. A gender-specific adaption of an existing intervention with potential initiators persistent in their efforts to obtain initiation and often fail to anticipate how injecting in front of non-injectors could lead to initiation requests. The intervention increases contemplation about injecting; reduce injecting in front of non-injecting drug users (NIDUs) and discussing injecting with NIDUs; increase disapproval of initiating others and increase competence in managing requests for initiation [10].

Second, a main finding of this study is that many women received their first injection from a female rather than a male sexual partner, running counter to literature claims that male partners initiate women to IDU [10-14,22-24]. However, it is somewhat consistent with the social or drug using network literature. Many of the women in this study were initiated to IDU by another woman were in a largely female drug using networks. This finding demonstrates the crucial role that WIDUs play in not only their transition from non-injection to IDU but also women’s initiation experience, both as influential peers and as injecting initiators of non-injecting women. Data consistently show that IDU is a highly gendered activity; it is surprising that much of the literature on IDUs does not distinguish between men and women IDUs. This may have led to an underrepresentation and clear need for new and more systematic collection of data specifically with WIDUS. For instance, there is new body of research that is showing that other females are initiating young non-injecting females [24-26]. This is an important area that requires further research. The author is currently conducting a quantitative study of WIDUs in NYC syringe exchange programs.

Another novel finding is that many of the women were self-injectors. These women exhibited decision-making and agency around the circumstances of injection safety and potential risks. They chose safer injection practices; carrying sterile syringes, preparing their own drugs, and not sharing prepared drugs or injecting equipment. Self-injectors avoided withdrawal and harm through self-reliance, injecting in private and safe venues, and participating in syringe exchange. This sense of autonomy and control highlights their lack of dependence on an injection partner. These findings are contrary to literature signifying that women’s drug initiation, continued access to drugs, and injecting occur mainly through male sexual partners. It is surprising that no prospective study has fully examined the protective factor of self-injection and it’s relation to HIV infection and transmission.

Fourth, in stark contrast, several women required assisted injection throughout their injecting career because of their lack of knowledge or deference to an injector due to anxiety or withdrawal. The presence of an established SEP did little to mitigate this risky behavior. This is concerning, as non-self injection is an independent predictor for HIV infection [1]. Women who require help with the injecting process are more likely to share syringes and injection equipment and have skin infections [27]. Sharing in the injection process and the subsequent high, some assisted injectors revealed their increased sense of trust and intimacy with their partner. Consequently, assisted injecting tended to be a symbolic act in the context of intimate relationships and represent an important point of intersection between sexual and injecting dynamics, comprising a dual risk for HIV acquisition [28,29].

One limitation to the research was the sampling method. Although this sample size was consistent with the qualitative research goal of gathering extensive information from a small group of individuals, the 26 women interviewed may not generalize to WIDUs who were not receiving services in a HRC or SEP. A second limitation was that respondent-driven sampling to recruit subjects was not used, and therefore the sample is not necessarily generalizable to the WIDU populations in SEPs in NYC. Nevertheless, saturation was observed on a number of themes, which provides confidence that the findings are meaningful. Additionally, the data was collected through the women’s self-report in a face-to-face interview format and the women may have been subject to providing socially desirable responses.

## Conclusions

Findings suggest that interventions that dissuade women who inject drugs from transitioning to IDU are to a great extent are needed and enhance self-efficacy and independence among women WIDUs. Development of peer-driven experiential interventions with strong female representation to dispel myths with the aim to dissuade women from transitioning to IDU and increase their personal capability to self-injection is needed. Interventions could provide information, enhance risk-reduction skills, and motivate injection practice change. WIDUs should always be involved in the design and implementation of these programs, to ensure that programs are effective, appropriate, and respectful of human rights [30]. Government and other policymaking bodies should strive to include WIDUs on relevant committees, involve them in hearings, and otherwise support substantive participation. Increase research funding to encourage systems to survey women’s injection practices are urgently needed to identify new trends in the number of WIDUs, their characteristics, and to improve and support women-specific evidence-based services.

## Abbreviations

IDU: Injecting drug use
WIDUs: Women injecting drug users
HRC: Harm reduction center
SEP: Syringe exchange program
